# DNMT3A reads and connects histone H3K36me2 to DNA methylation

**DOI:** 10.1007/s13238-019-00672-y

**Published:** 2019-11-22

**Authors:** Wenqi Xu, Jiahui Li, Bowen Rong, Bin Zhao, Mei Wang, Ruofei Dai, Qilong Chen, Hang Liu, Zhongkai Gu, Shuxian Liu, Rui Guo, Hongjie Shen, Feizhen Wu, Fei Lan

**Affiliations:** 1 Key Laboratory of Birth Defects, Children’s Hospital, Fudan University, and Key Laboratory of Epigenetics, Institutes of Biomedical Sciences, Fudan University, Shanghai, 201102 China; 2 Department of Geriatrics, Shanghai General Hospital, Shanghai, 201103 China; 3 Research Center for Chinese Traditional Medicine Complexity System, Shanghai University of Chinese Traditional Medicine, Shanghai, 201203 China

**Dear Editor,**

DNA methylation at the 5-position of cytosine (5mC) is a crucial epigenetic mark in regulating biological processes including gene silencing, gene imprinting, and X chromosome inactivation (Jaenisch and Bird, 2003; Smith and Meissner, 2013). Human genome encodes three DNA methyltransferases, DNMT1, DNMT3A and DNMT3B to catalyze 5mC. Although not tightly restricted, DNMT1 is thought to maintain the established pattern of 5mC throughout DNA replication, while DNMT3A and DNMT3B are largely responsible for the de novo establishment of 5mC. It has long been questioned how de novo DNA 5mC patterns are established in different genomic regions and whether histone modifications crosstalk to the process. Until recently, it was reported that through recognition of histone H3K36me3 mark, DNMT3B plays a dominant role in mediating DNA 5mC in the genic region undergoing active transcription (Baubec et al., 2015; Neri et al., 2017). However, 5mC occurs at both intergenic and genic regions, while H3K36me3 is largely absent in the intergenic regions, indicating that the intergenic 5mC may be mediated through different mechanisms.

Although H3K36me3 is involved in the DNMT3B mediated DNA 5mC in the genic regions (Baubec et al., 2015; Neri et al., 2017), we found that SETD2 loss in HEK293T cells did not cause reduction of global 5mC level, which was actually moderately increased by around 20% (Fig. S1A, left and middle), indicating alternative mechanism in directing DNA 5mC by histone modifications might exist. Unexpectedly, in the SETD2 KO HEK293T cells, we also observed an obvious increase of H3K36me2 (Fig. S1A, right). Such gain of H3K36me2 and increase of 5mC in the SETD2 KO HEK293T cells raised an interesting possibility of H3K36me2 in regulating DNA 5mC. To explore this possibility, we first compared the distribution patterns of 5mC with various well-characterized histone modifications, including H3K36me2, using public available datasets (Figs. 1A and S1B). Consistently, we observed a strong correlation between H3K36me2 and 5mC (*R* = 0.85) in MDA-MB-231 cells, which is even higher than that between H3K36me3 and 5mC (*R* = 0.63, Fig. 1A). The correlations between 5mC and H3K36me3 in 2 other cell lines are also around the same range (*R* = 0.64−0.66, Fig. S1B). Importantly, further genomic distribution analyses revealed that the similarity between 5mC and H3K36me3 is restricted to genic regions as reported previously (Baubec et al., 2015; Morselli et al., 2015), while that between 5mC and H3K36me2 is mainly in the intergenic regions and the immediate upstream to promoter regions in MDA-MB-231 cells (Fig. 1B, compare the blue and orange lines). Using the same approach, we found that H3K9me3 and H3K27me3 are poorly correlated with 5mC in MDA-MB-231 (Fig. S1C), indicating that the strong correlation between 5mC and H3K36me2 is rather specific.

**Figure 1:**
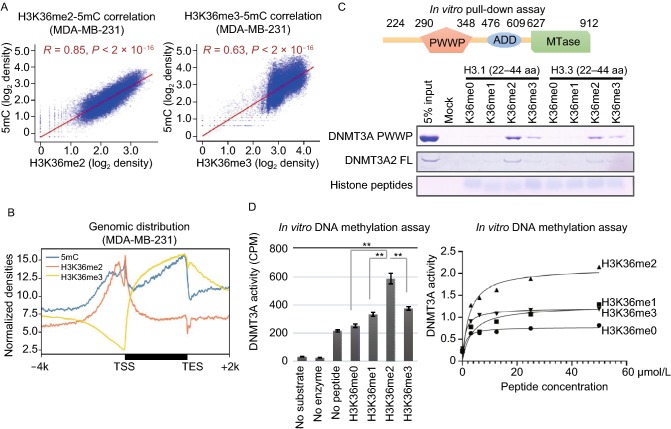
**Genome-wide correlation between DNA 5mC and H3K36me2 and DNMT3A preferentially binds and is activated by H3K36me2**. (A) Scatterplots showing genome-wide correlation between H3K36me2 and 5mC (left), and H3K36me3 and 5mC (right) in MDA-MB-231 cell. The number of reads was counted in nonoverlapping 100-kb bins spanning the human genome. The correlation coefficient was calculated with Pearson correlation. (B) Aggregation plot showing the distribution patterns of 5mC, H3K36me2 and H3K36me3 in the indicated genomic regions. (C) Schema of DNMT3A2 domain architecture and biotinylated peptide pull-down assay of DNMT3A2 FL and PWWP domain with the indicated histone H3.1K36 and H3.3K36 peptides. (D) *In vitro* DNA methylation assay testing the stimulation of DNMT3A activity with the indicated histone peptides (H3.1 22-44aa) (**P value < 0.01) (left, radioactivity), and at different concentrations (right, Michaelis-Menten graph analyses, MBD2b based)

Among the three DNA methyltransferases, both DNMT3A and DNMT3B possess a PWWP domain. As PWWP domain was reported to be capable in recognizing H3K36me2 or H3K36me3 (Sankaran et al., 2016), we hypothesized that the PWWP domain of DNMT3A may be able to read H3K36me2 and play a recruitment function for DNMT3A. Consistent with this idea, in vitro pull-down assay readily identified a specific interaction between H3K36me2 and the PWWP of DNMT3A (Fig. 1C). Interestingly, we found that the interaction was substantially stronger than that of H3K36me3 (Fig. 1C), and such preference was not affected by the sequence variation at the 31 position of the histone variants H3.1/2 and H3.3, despite the interactions with H3.3 peptides were generally weaker (Fig. 1C). Consistently, recombinant full length DNMT3A2 (isoform 2) purified from E.coli also preferentially binds H3K36me2 compared to H3K36me3 (Fig. 1C).

Various crosstalk mechanisms between histone modifications and 5mC have been documented, and it was known that the histone H3K4 methylations, especially H3K4me2/3, could strongly repress DNMT3A activity through ADD domain providing a mechanism to protect active promoters and enhancers from DNA 5mC methylation (Zhang et al., 2010; Li et al., 2011; Guo et al., 2015). We therefore wondered whether H3K36me2 binding by the PWWP domain could also affect the enzymatic activity of DNMT3A. To address this, we carried out in vitro methyltransferase assay using recombinant full length DNMT3A2 protein purified from insect cells and DNA substrate by spiking in H3K36me2 modified as well as several control histone peptides (Fig. 1D). In support of our hypothesis, we found that while H3K36me1 and H3K36me3 peptides only had minimal effects on DNMT3A activity, H3K36me2 peptides could significantly activate DNMT3A at a concentration as low as 3.2 μmol/L, using two independent, radioactivity and MBD domain-based, methylation detection methods (Fig. 1D).

In order to understand the biological impact of the axis of histone H3K36me2 and DNA 5mC, we turned to a well-characterized multiple myeloma model, KMS11 and KMS11^TKO^. KMS11 contains a chromosomal translocation event, T(4;14), which leads to massive overexpression of NSD2 and genome-wide gain of H3K36me2 (Kuo et al., 2011). While in KMS11^TKO^, the translocation event was specifically knocked out, resulting in monoallelic expression of NSD2 and close to normal level of H3K36me2 (Fig. 2A). Consistent with our in vitro results, both dot blot and quantitative HPLC analyses found that the extracted KMS11 genomic DNA contains around 30% more DNA 5mC compared to that of KMS11^TKO^, which is a significant alteration of global 5mC level (Fig. 2B).

**Figure 2:**
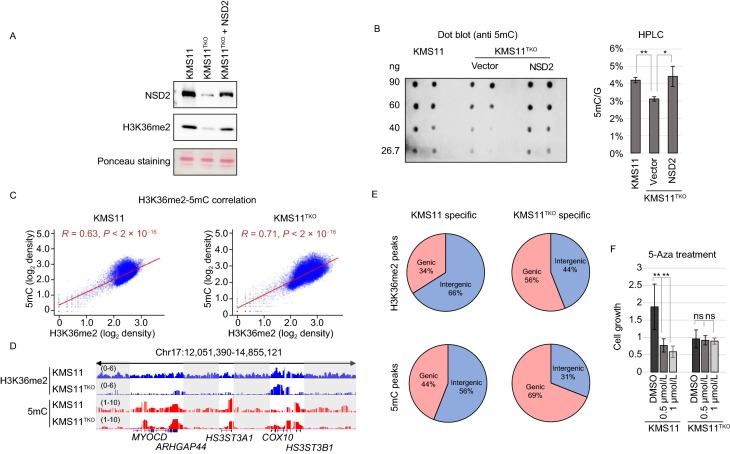
**Hyper DNA 5mC in KMS11 is dependent on NSD2 overexpression and is specific required for KMS11 growth**. (A) Western blot showing the expression level of NSD2 and H3K36me2 in the KMS11, KMS11^TKO^ and KMS11^TKO^+NSD2 (with NSD2 expression using a rescuing construct) cells. Ponceau staining of the histone region as loading control. (B) Global DNA 5mC analyses of the KMS11, KMS11^TKO^ and KMS11^TKO^+NSD2 cells using dot blot (left) and HPLC (right) approaches. **P < 0.01, *P < 0.05, *t*-test. (C) Scatterplots showing the correlation of H3K36me2 and 5mC in KMS11 and KMS11^TKO^ cells. (D) UCSC track snapshot showing the distribution patterns of H3K36me2 and 5mC in the indicated genomic region in KMS11 and KMS11^TKO^ cells. (E) Pie chart analyses of the genomic distribution of H3K36me2 (top) and 5mC (bottom) peaks specific to KMS11 or KMS11^TKO^ cells. (F) Cell growth analyses of KMS11 and KMS11^TKO^ cells under the treatment of 5-Aza at the indicated concentrations for 4 days (**P < 0.01, *t*-test)

Through MeDIP-seq and H3K36me2 ChIP-seq approaches in KMS11 and KMS11^TKO^ cells, we again observed strong correlations between H3K36me2 and DNA methylation genome-wide (Fig. 2C). Interestingly, the correlation is significantly higher in KMS11^TKO^ compared to KMS11 (Fig. 2C), indicating the abnormal hyper H3K36me2 significantly impaired proper 5mC establishment in KMS11. Close examination of mapped H3K36me2 and 5mC genome tracks found that the both marks are more dispersedly distributed in KMS11 genome due to genome-wide hyper H3K36me2 (Fig. 2D). While in KMS11^TKO^ cells, we found that most intergenic H3K36me2 signals were lost as the result of loss of NSD2 overexpression, and the remaining H3K36me2 signals were generally retained in the genic and surrounding regions, exemplified by *ARHGAP44* and *COX10* in Fig. 2D. Consistent with our hypothesis, we also observed significant reduction of the intergenic 5mC in KMS11^TKO^ (Fig. 2D, shadowed regions). Notably, genic 5mC signals were largely retained and in certain cases, such as *COX10*, even increased in KMS11^TKO^ (Fig. 2D), likely due to the effect of relatively enriched focal H3K36me2. We also noticed that, in KMS11^TKO^, not all genic 5mC signals matched H3K36me2 patterns (for examples, *HS3ST3A1* and *HS3ST3B1* in Fig. 2D), and we speculated that H3K36me3 might play a recruitment role in these regions. Consistent with the observations in Fig. 2D, the genomic distributions of KMS11-specific H3K36me2 and 5mC peaks are similar as average genome proportion (genic 45.3% and intergenic 54.7%), while KMS11^TKO^-specific peaks are more enriched in genic regions (Fig. 2E).

To further explore the functional importance on gene expression by the H3K36me2 and DNA 5mC axis, we carried out RNA-seq analyses in KMS11 and KMS11^TKO^ cells (Fig. S2A). As revealed by other studies (Kuo et al., 2011), we identified hundreds of differentially expressed genes (DEGs). Among these DEGs, we found that the up-regulated genes in KMS11 are significantly enriched for pathways in cancer (Fig. S2B), and the altered expression of several oncogenes in myeloma, such as *HSPG2*, *TREML2* and *NCAM1* were validated by RT-qPCR (Fig. S2C). We also found that the upregulated genes in KMS11 showed higher upstream DNA 5mC which tended to be lost in KMS11^TKO^, compared to the downregulated genes (Fig. S2D, left). While the 5mC levels of the CpG islands at the transcription start sites (TSSs) of the up- and down- regulated genes were generally low and similar, although statistically significant (Fig. S2D, right). This observation raises an intriguing possibility of the upstream hyper 5mC in KMS11 being functionally involved in the downstream gene transcription, which certainly needs future investigation.

As NSD2 overexpression was demonstrated as the driver mutation for multiple myeloma bearing T(4;14) translocation (Keats et al., 2003; Santra et al., 2003; Kuo et al., 2011), we therefore speculated that the gain of 5mC as the consequence of NSD2 overexpression may also functionally involved in the tumorigenic processes. Supporting this idea, we found that the application of DNA methylation inhibitor, 5-Aza, effectively suppressed the proliferation of KMS11 cells at 0.25 μmol/L in 4 days (Fig. 2F). While under same condition, even at 1 μmol/L concentration, 5-Aza showed no effect on the proliferation of KMS11^TKO^, indicating that the global hypermethylation of 5mC is required for cancerous growth driven by NSD2 overexpression in KMS11.

Our findings revealed a previously under-appreciated function of H3K36me2 in regulating DNMT3A mediated DNA 5mC. Although partially overlapped in genic regions with H3K36me3, H3K36me2 also demarcates many intergenic regions. Together with the fact that H3K36me3 directs genic DNA 5mC methylation through DNMT3B (Baubec et al., 2015), H3K36 methylations play critical role in guiding DNMT3A and DNMT3B to keep the proper level of DNA 5mC methylation genome-wide. Interestingly, both H3K36me2 and H3K36me3 are absent in the promoter regions, consistently with and perhaps also mechanistically contribute to the 5mC hypomethylation at the active promoters. Furthermore, as NSD family members frequently undergo gain-of-function mutations in MM, AML, lung and breast cancers (Rosati et al., 2002; Wang et al., 2007; Oyer et al., 2014), our findings also support therapeutic opportunities for DNA methylation inhibitors in treating these tumors.

## Electronic supplementary material

Below is the link to the electronic supplementary material.

Supplementary material 1 (PDF 385 kb)
