# Correction to: DNMT3A reads and connects histone H3K36me2 to DNA methylation

**DOI:** 10.1007/s13238-019-00678-6

**Published:** 2019-12-09

**Authors:** Wenqi Xu, Jiahui Li, Bowen Rong, Bin Zhao, Mei Wang, Ruofei Dai, Qilong Chen, Hang Liu, Zhongkai Gu, Shuxian Liu, Rui Guo, Hongjie Shen, Feizhen Wu, Fei Lan

**Affiliations:** 1 Key Laboratory of Birth Defects, Children’s Hospital, Fudan University, and Key Laboratory of Epigenetics, Institutes of Biomedical Sciences, Fudan University, Shanghai, 201102 China; 2 Department of Geriatrics, Shanghai General Hospital, Shanghai, 201103 China; 3 Research Center for Chinese Traditional Medicine Complexity System, Shanghai University of Chinese Traditional Medicine, Shanghai, 201203 China

## Correction to: Protein Cell 10.1007/s13238-019-00672-y

The author would like to add the below information in this correction.

A similar study from Chao Lu group was published online on 5 September 2019 in Nature, entitled “The histone mark H3K36me2 recruits DNMT3A and shapes the intergenic DNA methylation landscape” (Weinberg et al., 2019). Although both studies reported the preferential recognition of H3K36me2 by DNMT3A PWWP, ours in addition uncovered a stimulation function by such interaction on the activity of DNMT3A. On the disease connections, we used a NSD2 gain-of-function model which led to the discovery of potential therapeutic implication of DNA inhibitors in the related cancers, while the other study only used NSD1 and DNMT3A loss-of-function models.
