# Supplementary material for: DNMT3A reads and connects histone H3K36me2 to DNA methylation
Source: Protein Cell. 2019 Nov 22;11(2):150–4. doi: 10.1007/s13238-019-00672-y (PMC6954886; doi:10.1007/s13238-019-00672-y)

Methods

In vitro peptide pull-down assay

GST-DNMT3A2 full length (Figure 1C) and PWWP domain proteins were prepared from E. coli expression system, using pGEX-6p-1 vector and Rosetta strain. The transformants were grown at 37 °C till OD600 = 0.6, then were induced by 0.2 mM IPTG (isopropyl-β-D-thiogalactopyranoside) and further incubated at 16 °C overnight. The GST fusion proteins were purified using standard protocol (SMART-Lifesciences).

For in vitro pull-down assay, a total of 2 μg purified GST-DNMT3A2 FL or GST-DNMT3A PWWP proteins were incubated with 1 μg of biotinylated histone peptides in the binding buffer (50 mM Tris-HCl pH 7.4, 150 mM NaCl, 0.1% NP-40) at 4°C for 2 hours. The protein-peptide complexes were immobilized by streptavidin beads (SMART-Lifesciences) at 4℃ for 2 hours, and then washed 5 times with binding buffer. The bound proteins were finally separated by 10% SDS-PAGE followed by Coomassie Blue staining.

In vitro DNA methylation assay

For Figure 1D (Left), Sf9 purified DNMT3A2 was purchased from Active Motif Inc (Catalog No: 31406). Methylation assay was performed as previously described (Guo et al.,2015). Briefly, 0.3 μM DNMT3A2 was incubated with 100ng DNA substrate (EBNA fragment:1.2 kb, including 52 CGs) in the reaction buffer (2.5 μM [methyl-^{3}H] AdoMet, 25 mM Tris–HCl pH 7.5, 5% glycerol, 0.01% 2-mercaptoethanol, 0.5 mg/ml BSA) for 30 min with addition of 3 μM histone peptides. The DNA products were extracted after Proteinase K digestion and subjected to liquid-scintillation counting (PerkinElmer). Each reaction was performed in triplicate.

For Figure 1D (Right), the in vitro DNA methylation assay was carried out with DNMT Activity / Inhibition Assay Kit from Active motif (Catalog No: 55006) following the manufacturer's protocol.

Dot blot and HPLC

Genomic DNA samples were extracted using DNeasy Blood and Tissue Kit (Qiagen, Catalog No:69506). For dot blot, DNA samples were first denatured at 98°C for 10 minutes and immediately cooled down in ice and then spotted on nitrocellulose membrane (Waterman) in serial dilutions of 1.5 fold. After ultraviolet crosslinking, membranes were blocked by 5% milk in TBST (10 mM Tris pH 7.4, 150 mM NaCl, and 0.1% Tween 20) at room temperature for 30 min and further incubated with 5mC antibody (Active Motif, catalog No:39649) at 4°C overnight, and developed using regular HRP conjugate secondary antibody.

For HPLC, the denatured DNAs were digested to single nucleosides using 0.5 U nuclease P1 (Sigma Aldrich: N3755-50UN) at 37°C for 16 hr and 0.5 U CIP (NEB:M0290) at 37°C for 1.5 hr. The samples were subjected to LC-MS/MS using a Shimadzu LC (LC-20AB pump) system.

ChIP-seq and MeDIP-seq and data analyses

ChIP and ChIP-seq assays were carried out as previously described (Shen et al.,2016) and the comparative MeDIP-seq was performed according to Li Tan, 2013.(Tan et al.,2013)

The ChIP-seq and MeDIP-seq reads were mapped to the human genome (hg19) using Bowtie (v1.2.2), duplicated reads were removed and only the unique mapped reads were kept for further analyses.

For ChIP-seq and MeDIP-seq analyses, peaks were identified by MACS1.4 with broad peak mode and other default parameters at cutoff of P value ≤ $1*10^{-5}$, FDR≤0.01. For comparison of H3K36me2 ChIP-seq data between the KMS11 and $KMS11^{TKO}$ cells, signal densities were normalized by total reads. The genomic distribution plot (Fig. 1A) was drawn with the deeptools.

The ChIP-seq and RNA-seq generated by this study have been deposited to GEO

database under accession number GEO: GSE140305. The public ChIP-seq and MeDIP-seq data used in this paper were listed below.

Cell type	histone modifications	MeDIP-seq
MDA-MB-231	SRR594894(H3K36me2)<br>SRR6310000(H3K36me3)<br>SRR8370262(H3K9me3)<br>SRR8370264(H3K27me3)	SRR3362402
Peripheral Blood Mononuclear Primary Cells	GSM613880(H3K36me3)	GSM613911
Breast Myoepithelial Cells	GSM613873(H3K36me3)	GSM613857

mRNA-seq:

mRNA-seq were carried out according to manufacturer's guidelines (C-10365; Life Technologies) and our previous study (Shen et al., 2016). Briefly, the mRNA-seq reads were mapped to human genome (hg19) using the TopHat (v2.1.1 Trapnell et al.,2009) with default parameters. Differentially expressed genes were identified by the Cuffdiff in Cufflinks package (v2.2.1) at cutoffs of FPKM ≥ 0.1, Q value < 0.01, and fold change ≥ 2. GO analysis was performed with DAVID Bioinformatics Resources 6.8 Functional annotation tools.

References:

Guo, X., Wang, L., Li, J., Ding, Z., Xiao, J., Yin, X., He, S., Shi, P., Dong, L., and Li, G., et al.(2015). Structural insight into autoinhibition and histone H3-induced activation of DNMT3A. NATURE 517, 640-644.

Shen, H., Xu, W., Guo, R., Rong, B., Gu, L., Wang, Z., He, C., Zheng, L., Hu, X., and Hu, Z., et al.(2016). Suppression of Enhancer Overactivation by a RACK7-Histone Demethylase Complex. CELL 165, 331-342.

Tan, L., Xiong, L., Xu, W., Wu, F., Huang, N., Xu, Y., Kong, L., Zheng, L., Schwartz, L., and Shi, Y.,

et al.(2013). Genome-wide comparison of DNA hydroxymethylation in mouse embryonic stem cells and neural progenitor cells by a new comparative hMeDIP-seq method. NUCLEIC ACIDS RES 41, e84.

Trapnell, C., Pachter, L., and Salzberg, S.L.(2009). TopHat: discovering splice junctions with RNA-Seq. BIOINFORMATICS 25, 1105-1111.

Supplementary Figure

Figure S1 Related to Figure 1

(A) Western blot analyses of SETD2 and H3K36me3 levels (left), HPLC analysis of the global DNA 5mC levels (middle), and quantitative histone mass spectrometry analysis of the H3K36 methylation levels in the control and SETD2 KO HEK293T cells (right). ** $p < 0.01$, * $p < 0.05$, t-test

(B-C) Scatterplots showing genome-wide correlation between the indicated histone marks and 5mC in (B) PBMP and Breast Myoepithelial, and (C) MDA-MB-231 cells. The number of reads was counted in nonoverlapping 100-kb bins spanning the whole genome. The correlation coefficient was calculated with Pearson correlation.

Figure S2 Related to Figure 2

(A) Vocanol plot showing the differentially expressed genes in KMS11 and KMS11^{TKO} cell lines.

(B) KEGG analysis showing the Top 5 enriched pathways for the differentially expressed genes in KMS11 compared to KMS11^{TKO}.

(C) RT-qPCR validation of the significant upregulation of *HSPG2*, *TREML2* and *NCAM1* in KMS11 compared to KMS11^{TKO} cells (** $p < 0.01$, t-test).

(D) Boxplots showing the 5mC difference between KMS11 and KMS11^{TKO} at gene upstream regions (-2k to -8k) (left) or gene promoter CGI regions (right).

Figure S1

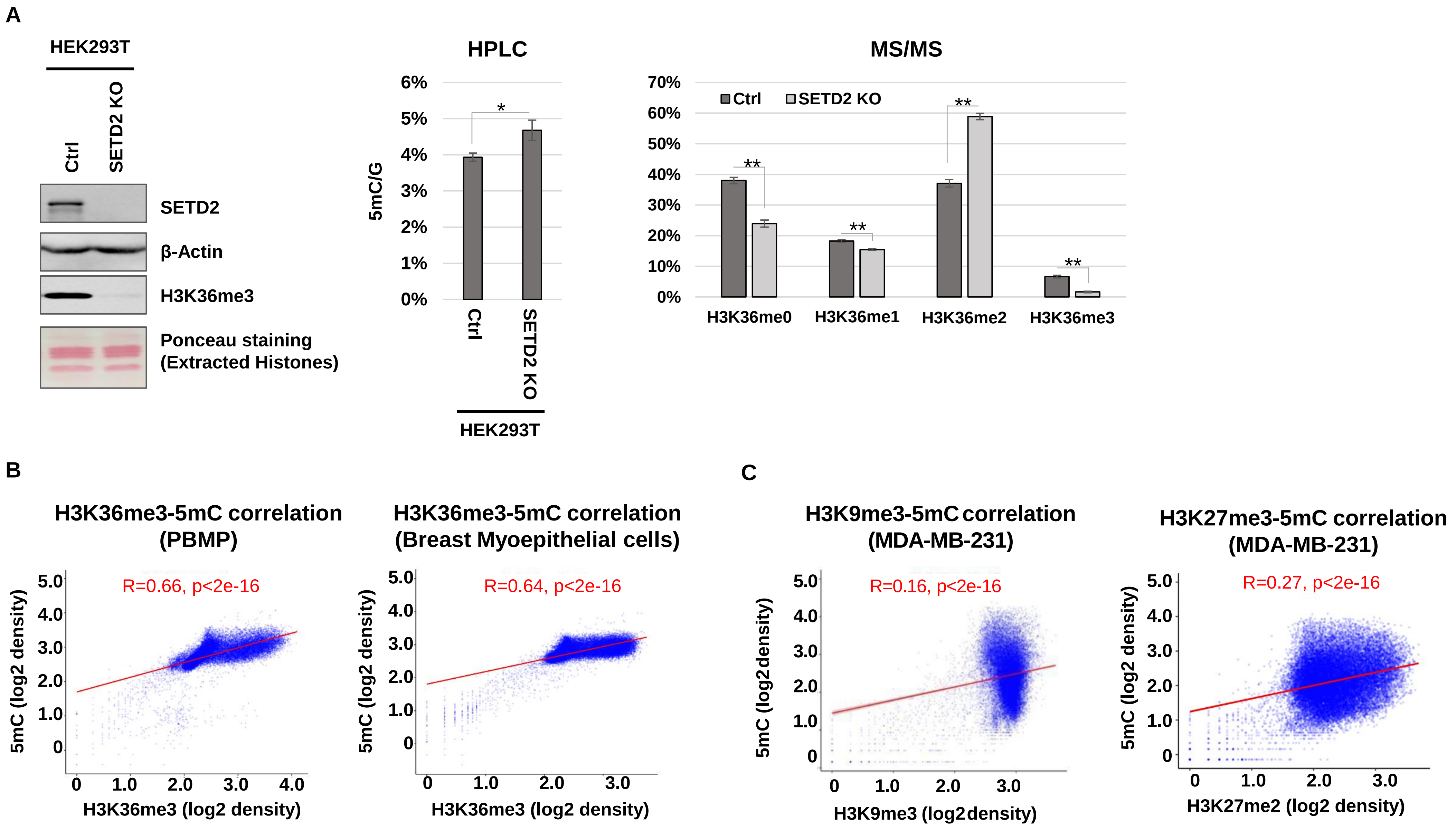
A
HEK293T
Ctrl
SETD2 KO
SETD2
β-Actin
H3K36me3
Ponceau staining
(Extracted Histones)
HPLC
5mC/G
6%
5%
4%
3%
2%
1%
0%
*
Ctrl
SETD2 KO
HEK293T
MS/MS
70%
60%
50%
40%
30%
20%
10%
0%
Ctrl
SETD2 KO
**
H3K36me0
H3K36me1
H3K36me2
H3K36me3
B
H3K36me3-5mC correlation
(PBMP)
R=0.66, p<2e-16
5mC (log2 density)
H3K36me3 (log2 density)
H3K36me3-5mC correlation
(Breast Myoepithelial cells)
R=0.64, p<2e-16
5mC (log2 density)
H3K36me3 (log2 density)
C
H3K9me3-5mC correlation
(MDA-MB-231)
R=0.16, p<2e-16
5mC (log2density)
H3K9me3 (log2density)
H3K27me3-5mC correlation
(MDA-MB-231)
R=0.27, p<2e-16
5mC (log2 density)
H3K27me2 (log2 density)

Figure S2

A

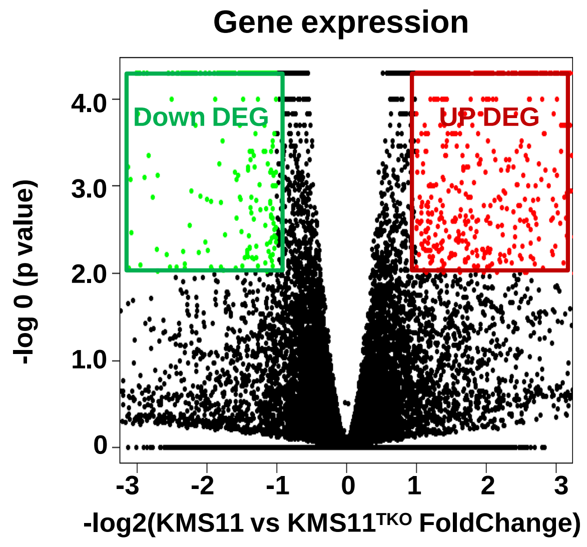

B

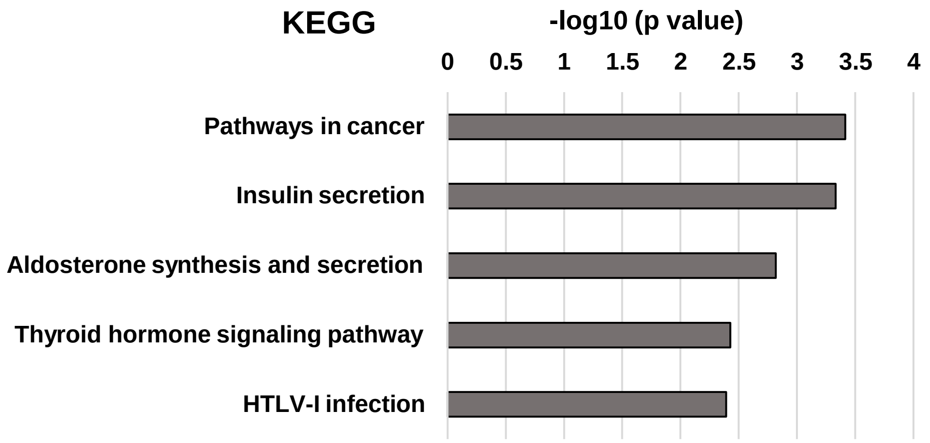

C

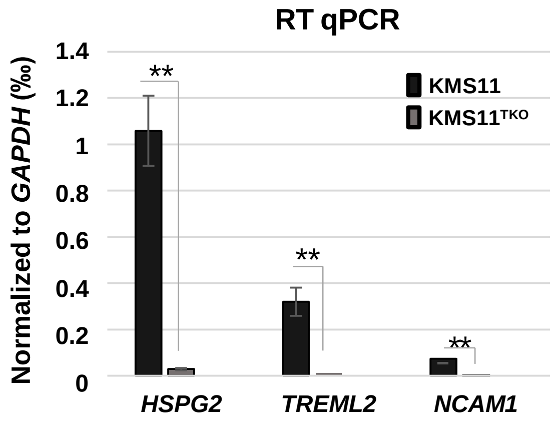

D

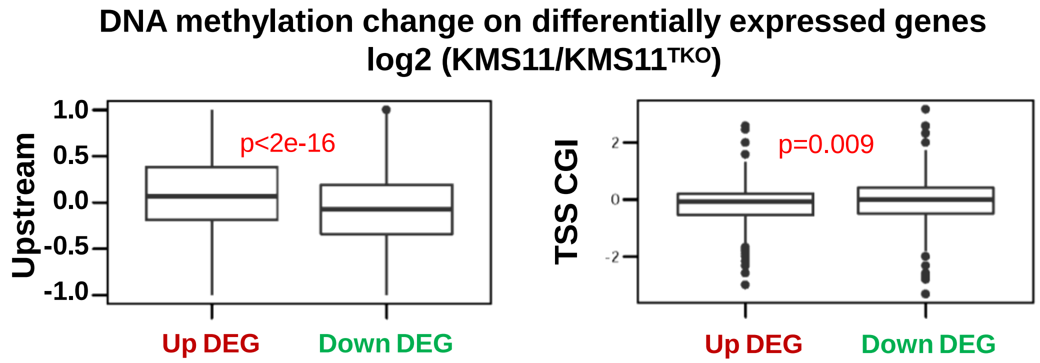

Supplement: Supplementary file 1 — Supplementary material 1 (PDF 385 kb) [file 13238_2019_672_MOESM1_ESM.pdf]
